# Individual-to-Resource Landscape Interaction Strength Can Explain Different Collective Feeding Behaviours

**DOI:** 10.1371/journal.pone.0075879

**Published:** 2013-10-09

**Authors:** Nikolai W. F. Bode, Johann Delcourt

**Affiliations:** 1 Department of Mathematical Sciences, University of Essex, Colchester, United Kingdom; 2 Behavioral Biology Unit: Ethology and Animal Psychology, Laboratory of Fish and Amphibian Ethology, Department of Environmental Sciences and Management, Faculty of Sciences, Institute of Zoology, University of Liège, Liège, Belgium; Institut Pluridisciplinaire Hubert Curien, France

## Abstract

Taking in sufficient quantities of nutrients is vital for all living beings and in doing so, individuals interact with the local resource environment. Here, we focus explicitly on the interactions between feeding individuals and the resource landscape. In particular, we are interested in the emergent movement dynamics resulting from these interactions. We present an individual-based simulation model for the movement of populations in a resource landscape that allows us to vary the strength of the interactions mentioned above. The key assumption and novelty of our model is that individuals can cause the release of additional nutrients, as well as consuming them. Our model produces clear predictions. For example, we expect more tortuous individual movement paths and higher levels of aggregation in populations occupying homogeneous environments where individual movement makes more nutrients available. We also show how observed movement dynamics could change when local nutrient sources are depleted or when the population density increases. Our predictions are testable and qualitatively reproduce the different feeding behaviours observed in filter-feeding ducks, for example. We suggest that considering two-way interactions between feeding individuals and resource landscapes could help to explain fine-scale movement dynamics.

## Introduction

Taking in sufficient quantities of nutrients is vital for all living beings. In doing so, individuals typically reduce local nutrient availability and thereby affect the local resource environment. For example, sheep prefer grass over heather but lack of, or depletion of the former can result in widespread defoliation of the latter [1]. Likewise, the behaviour of individual organisms is affected by the distribution of nutrients. To give an example, it has long been hypothesized and is still debated whether the movement patterns of individual animals optimises their chances of finding food in patchy resource distributions [2]–[4].

Aggregations of individual organisms are commonplace in nature. The reasons for animals to aggregate depend on the context and while most aggregations are believed to be formed in response to predation, higher local nutrient availability or improved ability to find nutrients provide alternative mechanisms [5]–[7]. There is no doubt that many aggregations in which individuals are consuming nutrients can be observed. Particularly striking examples of this scenario are aggregations displaying collective behaviour - distinct aggregation-level phenomena that emerge from individual actions [8]. One famous example is the movement of swarms of locusts consisting of thousands of individuals that do not disperse, but maintain a certain degree of cohesion and directionality and devastate all vegetation in their path [9].

Individuals may derive various benefits from collective behaviours in feeding aggregations. Being part of an aggregation may reduce the likelihood of individuals being predated [5], [10]–[12] and collective behaviour may lead to an improvement over individual abilities in finding sources of nutrients or nutrient-rich locations [13]. A particularly advantageous effect of collective behaviour for individuals in groups is the improved ability or efficiency to exploit nutrient resources. For example, many predators work in groups to be able to tackle prey they could not hunt individually [14], [15]. Another well-known example is given by colonies of eusocial insects. Pheromone trails laid and re-enforced by ants returning to the nest [16], [17] and “waggle-dances” of bees in the hive [18] indicate the location of food sources to other workers and increase the efficiency of their exploitation.

In the first paragraph, we briefly introduced the interactions of feeding individuals with their resource or nutrient environment. The preceding discussion illustrates that aggregations or groups react to the distribution of nutrients by laying a particular network of pheromone trails or by using collective sensing to find nutrient-rich locations, for example. Interestingly, feeding animal groups can also impact on the resource landscape beyond simply depleting it. Experiments suggest that mobile schools of tadpoles can enhance their foraging success by disturbing the substratum of ponds through their movement which frees additional nutrients [19], [20]. More specifically, experiments have shown that tight vortex-like formations of tadpole schools affect the distribution of nutrients [20] and that social interactions result in increased growth in tadpoles, possibly related to increased nutrient intake [21]. It has been suggested that this schooling behaviour in tadpoles could represent a collective foraging strategy [19], [20]. Individual animals have also been shown to affect the resource landscape beyond depletion. For example, experiments suggest that the swirling movement of wadepipers (*Phalaropus sp*., Scolopacidae) in shallow water helps to raise prey items to the surface and therefore within reach of the birds [22]. Similar mechanisms have been suggested for ducks filtering plankton from the water [23]–[25].

We focus explicitly on the interactions between feeding individuals and the resource landscape. These interactions have been the subject of previous theoretical work. For example, models have helped to explain how ants explore or exploit their environment by laying and reacting to trails of pheromones [16], [17] and Czirok and co-workers [26] presented a detailed individual-based model for the formation of bacterial colonies in a resource landscape. Here, we are interested in the emergent movement dynamics resulting from individual-to-resource landscape interactions. We present a simulation model for the movement of individuals in a resource landscape that allows us to vary the strength of the above mentioned interactions. The key assumption and novelty of our model is that individuals can cause the release of additional nutrients, as well as consuming them. In this way, individuals leave a trace in the environment that affects the actions of other individuals, thereby leading to indirect interactions via the resource landscape. This presents a mechanism of indirect coordination between individuals, a concept called ‘stigmergy’ [27].

In our model, we restrict social interactions to collision avoidance and show that indirect interactions via the nutrient field can result in varied movement dynamics, such as dispersed feeding and near linear movement of individuals or aggregations with vortex-like internal dynamics. Previous theoretical work has shown that varied group level movement dynamics can occur as a result of changes in the balance of social interactions between individuals, such as the tendency to align or move towards other group members (e.g. [28]). Our simulations show that different collective behaviours including aggregation or vortex-like dynamics need not be the result of changes in direct social interactions, but can arise from changes in individual interactions with the resource landscape alone. Our approach results in testable hypotheses for the movement dynamics expected in feeding populations occupying resource landscapes with different characteristics and our results qualitatively match different feeding behaviours observed in filter-feeding ducks, for example.

## Model

In the following, *|* ***a*** *|* denotes the norm or length of a vector ***a***. We implemented our model in the JAVA programming language (http://www.java.com/).

### Individual Behaviour

The individual-based model simulates movement in two continuous spatial dimensions within a toroidal box of side length *L* (periodic boundary conditions; if an individual crosses one boundary, it appears on the opposite side of the box). We simulate a total of *N* individuals and all individuals *i,* where *i = 1, 2, …, N*, update their position ***x_i_*** and velocity ***v_i_*** at fixed time steps of length Δ*t = 0.1* seconds according to the following rules.
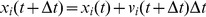
(1)

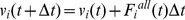
(2)


The quantity ***F_all_(*** *t* ***)*** describes the force acting on each individual at time *t*. This force sums up the behaviour and effect of the environment on individuals,
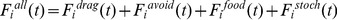
(3)


The force, ***F_i_^drag^(*** *t* ***)*** *= -γ* ***v_i_(*** *t* ***)*** implements friction in the system. A degree of uncertainty is added to individuals’ movement via ***F_i_^stoch^(*** *t* ***)***, a vector with its length chosen from a uniform distribution over *[0, ξ]*, pointing in a direction chosen at random from between *0* and *2π*. The forces ***F_i_^avoid^(*** *t* ***)*** and ***F_i_^food^(*** *t* ***)*** implement individuals’ interactions with others (short range) and interactions with the environment (seeking nutrient-rich regions), respectively, and will be defined in more detail below.

The only direct interaction between individuals is a short-range repulsion which could be interpreted as individuals’ tendency to avoid collisions or prolonged contact with others. Based on these considerations, we set
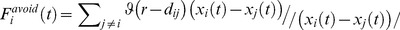
(4)where *d_ij_* is the Euclidean distance between individuals *i* and *j* and the Heaviside function *θ* implements the active interaction range, *r*. We deliberately only include this type of social interaction, as we are primarily interested in how interactions of individuals with the environment give rise to varying movement dynamics.

We focus on one aspect of the environment, namely the availability of nutrients. Individuals seek nutrient rich regions throughout our simulations – we assume that nutrient uptake is low compared to the energy demand of individuals. The force acting on individuals as a result of interacting with the environment, ***F_i_^food^(*** *t* ***)***, has two components, ***f_i,1_*** *(t)* and ***f_i,2_*** *(t)*. We set ***F_i_^food^(*** *t* ***)*** *=* ***f_i,1_*** *(t)+* ***f_i,2_*** *(t)*. The first component, ***f_i,1_*** *(t)*, is of unit length and points in the direction of the highest nutrient gradient within the nutrient-specific sensory range, *R*, of individuals. We defer the details of how the highest gradient is determined to the next section when we will explain the implementation of the dynamics in the environment. The second component of ***F_i_^food^(*** *t* ***)***, ***f_i,2_*** *(t)*, is an additional propulsion term, also of unit length, that points in the direction of ***v_i_(*** *t* ***)*** if the scalar product between ***v_i_(*** *t* ***)*** and ***f_i,1_*** *(t)* is positive. If this scalar product is negative, ***f_i,2_*** *(t)* points in the direction orthogonal to ***v_i_(*** *t* ***)*** that minimizes the angle between ***f_i,1_*** *(t)* and ***f_i,2_*** *(t)*. The rationale for this implementation is that ***f_i,2_*** *(t)* represents a self-propulsion term for individuals. We assume that the way in which individuals feed requires them to maintain a certain speed and not slow down to a halt. Thus, when the highest nutrient gradient has a component opposite to the movement direction of individuals, we use the propulsion term to enforce a turn towards this nutrient gradient, as opposed to slowing down and then reversing the movement direction. Furthermore, note that ***f_i,1_*** *(t)* is a unit vector and does not depend on how steep the detected nutrient gradient is. We choose this parsimonious implementation as it is not clear whether animals detect and memorize absolute differences in nutrient gradients.

### Environment Dynamics

While individual movement occurs in continuous two-dimensional space, we discretise the simulation space of *LxL* units^2^ into a square grid of separate *0.1x0.1* units^2^ cells to simulate the dynamics of the nutrient field. For example, the nutrient field for a *5x5* units^2^ simulation space consists of a square grid of *50x50* cells. We found this level of resolution to be sufficient for our purposes.

The main novelty of our work is that we consider not only the reaction of individuals to the nutrient field, but also the impact of individuals on the nutrient field. For this purpose, we simulate two nutrient fields. The first one is directly available to foraging individuals and denoted by *Q_k,l_(t)*. The other nutrient field, *U_k,l_(t)*, encapsulates the underlying distribution of nutrients that are not directly available to foraging individuals. The indices *k* and *l* indicate the grid cells and run from *1* up to *10 L*.

We assume that the movement of individuals disturbs the underlying nutrient field and frees additional nutrients. For example, the movement of ducks on a shallow lake could perturb the sediment at the bottom of the lake and bring plankton or small insects up to the surface where the ducks can filter these nutrients out of the water [29]. Furthermore, we assume that the faster individuals move, the more additional nutrients they can free, as formalized below.

Based on these considerations, we simulate the dynamics of the two nutrient fields according to the following equations:
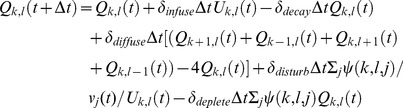
(5)

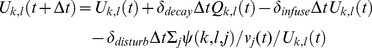
(6)


The function *ψ(k,l,j)* takes value one if individual *j* is positioned on the grid cell *(k,l)* and equals zero otherwise.

At a rate controlled by *δ_infuse_*, nutrients become available from *U* into *Q* and at a rate controlled by *δ_decay_*, nutrients become unavailable and settle back from *Q* into *U* (second and third terms in equation 5). Within *Q*, we implement a simple form of nutrient diffusion that is controlled by the parameter *δ_diffuse_* (fourth term in equation 5). Individuals moving over the nutrient field disturb *U* and thereby locally add additional nutrients to *Q* at a rate controlled by the parameter *δ_disturb_* and their movement speed (fifth term in equation 5). Finally, individuals consume available nutrients from *Q* at a rate controlled by *δ_deplete_* (final term in equation 5). Nutrient consumption is proportional to the available nutrients and does not saturate as we assume that consumption remains well below the saturation point for individuals. In the rare case when equations 5 and 6 would result in values of *Q* or *U* smaller than zero, we distribute the available quantity of nutrients over the terms in the equations according to their relative weighting and set the value of the corresponding nutrient cell to zero. However, we choose small parameter values for the rates discussed above so that this case rarely occurs in practice.

To complete the description of our model, we specify how individuals compute the direction of the highest gradient in the nutrient field *Q*. We denote the unit vector pointing in the direction from the midpoint of the grid cell *(k,l)* towards the midpoint of the grid cell *(m,o)* in *Q* on continuous space by ***q_k,m,l,o_***. Furthermore, we assume individual *i* is positioned on grid cell *(k,l)* at time *t*. Then, the vector ***f_i,1_*** *(t)* is the unit vector pointing in the direction given by the following vectorial sum *Σ_m = k-R…k+R_ (Σ_o = l-R…l+R_ [Q_m,o_(t) - Q_l,k_(t)]* ***q_k,m,l,o_*** *)* unless the sum equals *(0,0)*. This case occurs if all the vectors ***q_k,m,l,o_*** cancel or the nutrient field *Q* is homogeneous and then the vector ***f_i,1_*** *(t)* is set to *(0,0)*.

All model parameters are listed and briefly described in table 1.

**Table 1:** List of model parameters.

Parameter	Values explored	Description
*N*	*20–500*	Number of individuals in the population.
*L*	*10 units*	Side-length of simulated space.
Δ*t*	*0.1 s*	Length of time-step between model updates.
*γ*	*1.2 s^−1^*	Friction coefficient.
*ξ*	*1.0 units*	Maximum length for stochastic component of individual behaviour.
*r*	*0.3 units*	Interaction radius for short range repulsion between individuals.
*R*	*0.5 units*	Sensory range over which individuals detect nutrient field gradients.
*δ_infuse_*	*0.001 s^−1^*	Controls the rate at which nutrients become available from *U* into *Q*.
*δ_decay_*	*0.1 s^−1^*	Controls the rate at which nutrient decay from *Q* into *U*.
*δ_deplete_*	*0–1 s^−1^*	Controls the rate at which individuals deplete local nutrients (removed from system)
*δ_diffuse_*	*0.001 s^−1^*	Controls the rate at which nutrients diffuse in *Q*.
*δ_disturb_*	*0–1 s^−1^*	Strength of the impact individual movement has on making nutrients available from *U* into *Q*.

Brief descriptions and values explored for all model parameters.

### Simulations and Initial Conditions

For each of our simulations we start individuals from newly generated random initial positions in the environment and with velocities of unit length pointing in a randomly chosen direction.

The initial nutrient distribution in *U* is obtained in one of two ways. First, we generate a completely homogeneous nutrient distribution by allocating ten food units to each grid cell, resulting in a total of *10(10 L)^2^* food units. We use this homogeneous nutrient distribution in all but one illustrative simulation. Second, we concentrate *10(10 L)^2^* food units around one randomly chosen x,y coordinate on the grid. Around this patch centre, we add food units by drawing two values from a normal distribution with mean zero and standard deviation *1* for each food unit added. We round these values to integers and add them to the grid coordinates of the patch centre to determine the position on the grid where the food unit is added.

Initially, all cells of the available nutrient field *Q* have value zero. The periodic boundary conditions are observed for both nutrient field dynamics and nutrient field generation. Simulations last for *300 seconds* ( = 3000 simulation steps) and we only analyse the dynamics of the last *50 seconds* of our simulations. From our simulations, we record at each time step the position and nutrient consumption for all individuals.

From the initial conditions we first update the nutrient fields and subsequently the positions and movement of all individuals and continue to update the nutrient fields and individual movement in this order throughout our simulations.

### Data Analysis

We compute six summary statistics that quantify the characteristics of the emergent movement dynamics.

#### Number of groups

We define groups as individuals that are connected either directly or indirectly via other connected individuals. Individuals are considered to be connected if they can interact socially. In our case this is when individuals are within their interaction range, *r = 0.3 units*. The number of groups is a measure for the degree to which the population aggregates. Low numbers of groups indicate that the population is aggregated, whereas high numbers indicate that the population is dispersed.

#### Size of the largest group

Based on the definition for groups above, we find the size of the largest group in the population. This statistic complements the number of groups in measuring the extent to which the population is aggregated. We allow this statistic to take value one if all individuals are isolated.

#### Polarisation

This commonly used summary statistic [28], [30] quantifies the degree to which all individuals move in the same direction and is computed as *| {Σ_j = 1_^N^ (* ***x_j_(*** *t* ***)*** *–* ***x_j_(*** *t-*Δ*t* ***)*** *)/|(* ***x_j_(*** *t* ***)*** *–* ***x_j_(*** *t-*Δ*t* ***)*** *)|} |/N* taking values between one (high alignment) and zero (low alignment). We typically only compute this statistic for the largest group in the population (i.e. we restrict the index *j* above to members of this group). If we do so, we require the largest group to contain at least two individuals.

#### Angular momentum

This statistic quantifies the degree of rotation of groups around the centre of the group, taking Values between one (high degree of rotation) and zero [28]. We only compute this statistic for the largest group in the population and require this group to contain at least two individuals. Let *N_l_* be the size and let the index *j* run over members of this group. Further, let ***c_l_*** *= Σ_j_* ***x_j_(*** *t* ***)*** */N_l_*, be the centre of this group and denote ***r_jc_(*** *t* ***)*** *= (* ***x_j_(*** *t* ***)*** *–* ***c_l_*** *)/|(* ***x_j_(*** *t* ***)*** *–* ***c_l_*** *)|* and ***s_j_(*** *t* ***) =*** *(* ***x_j_(*** *t* ***)*** *–* ***x_j_(*** *t-*Δ*t* ***)*** *)/|(* ***x_j_(*** *t* ***)*** *–* ***x_j_(*** *t-*Δ*t* ***)*** *)|*. Then the angular momentum is given by *|Σ_j_* ***r_jc_(*** *t* ***)*** *x* ***s_j_(*** *t* ***)*** *|/N_l_*, where we use ***x*** *x* ***y*** *= (x_1_y_2_ - x_2_y_1_)*.

### Tortuosity

As a measure of how direct the movement of individuals is, we compute the ‘arc-chord’ ratio: the total distance an individual moves in *2 seconds* of simulation time divided by the distance between the positions of this individual at the start and the end of the 2 *second* interval. Values close to one indicate that individuals move in a straight line and the larger the tortuosity, the less direct is the movement of individuals. We report the average tortuosity across individuals.

### Consumption

We report the average nutrient consumption of individuals by the sum over the field of the last term in equation 5, divided by *N*. We normalise this absolute consumption by dividing it by the value of *δ_deplete_* to obtain the consumption relative to the feeding capacity. If *δ_deplete_ = 0*, we set the normalised consumption to zero.

Unless otherwise stated, we compute the average for each summary statistics over the last *50 seconds* of simulation time for each simulation.

## Results

**Figure 1:**
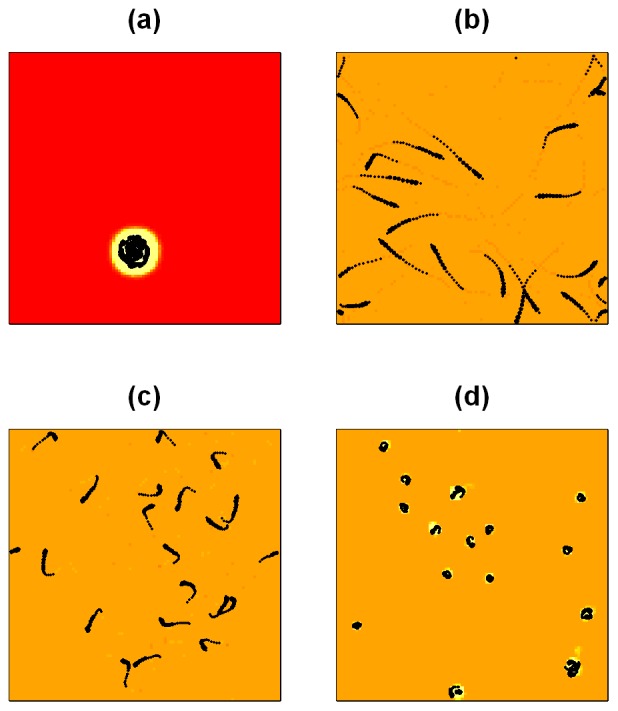
Illustration of different group dynamics obtained from simulations of 20 individuals. We show the distribution of available nutrients, *Q*, at the end of a *20 second* simulation. Brighter colours correspond to higher nutrient concentrations in *Q*. The colouring scheme is consistent across a-d and we have not included a scale, as nutrient levels are measured in arbitrary units. Individual trajectories over the last *1.5 seconds* of the simulation are shown in black. (a) ‘vortex’ around an area of high underlying nutrient concentration, *(δ_deplete_, δ_disturb_) = (1, 0.1)*. In (a), the nutrients in *U* are initially concentrated in one patch. (b)-(d) have initially homogeneous underlying nutrient fields *U*. (b) no release of nutrients through individuals’ movement, *(δ_deplete_, δ_disturb_) = (1, 0)*. (c) weak release of nutrients through individuals’ movement, *(δ_deplete_, δ_disturb_) = (1, 0.01)*. (d) ‘swirling individuals’, *(δ_deplete_, δ_disturb_) = (1, 0.1)*. All other parameter values are given in table 1.

Figure 1a shows that groups form stable vortices around an area of high nutrient concentration (see also film S1). The high concentration of nutrients attracts individuals and repulsive forces between individuals, as well as the turning behaviour towards high nutrient concentrations (see ***f_i,2_*** *(t)* above), cause the aggregation and circular motion of individuals in our simulations. Previous theoretical work has already demonstrated that self-propelled individuals readily form vortices around attractive potentials, even with minimal interactions between individuals [26], [31]. This result serves to illustrate that non-homogeneous nutrient distributions can affect the movement dynamics and we further discuss this below. However, here we investigate how the interactions of individuals with the nutrient fields impact on the emergent movement dynamics. To do so, we use initially homogeneous nutrient distributions *U* and vary the two parameters which quantify the interactions of individuals with the nutrient fields, *δ_deplete_* and *δ_disturb_*. The former parameter captures how quickly and completely individuals deplete available nutrients. The latter parameter captures the ability of individuals to free previously unavailable nutrients. This could be related to aspects of the physiology of organisms, but we suggest that it could capture properties of the environment. For example, in a shallow pond individuals swimming on the water surface may disturb the substratum more and thus free more nutrients than in a deeper pond.

Figure 1b-d illustrates that for initially homogeneous underlying nutrient distributions, a wide variety of behaviours can be obtained simply by varying the strength of interactions between individuals and the resource landscape (varying *δ_deplete_* and *δ_disturb_*). If individuals do not release additional nutrients through their movement (*δ_deplete_* = 1, *δ_disturb_ = 0*), their motion is approximately linear and only interrupted by repulsive interactions with other individuals (fig. 1b). As individuals release more nutrients through their movement, *δ_disturb_>0*, their trajectories become less linear (fig. 1c) up to the point when they move in a tight circle around a near stationary location (fig. 1d). These differences in behaviour are also reflected in the speed time-series of individuals and in the distribution of individual speeds across the population (figure S1). For a more detailed illustration of the dynamics described here, we refer the reader to the supplemental films (see films S2–S4).

**Figure 2:**
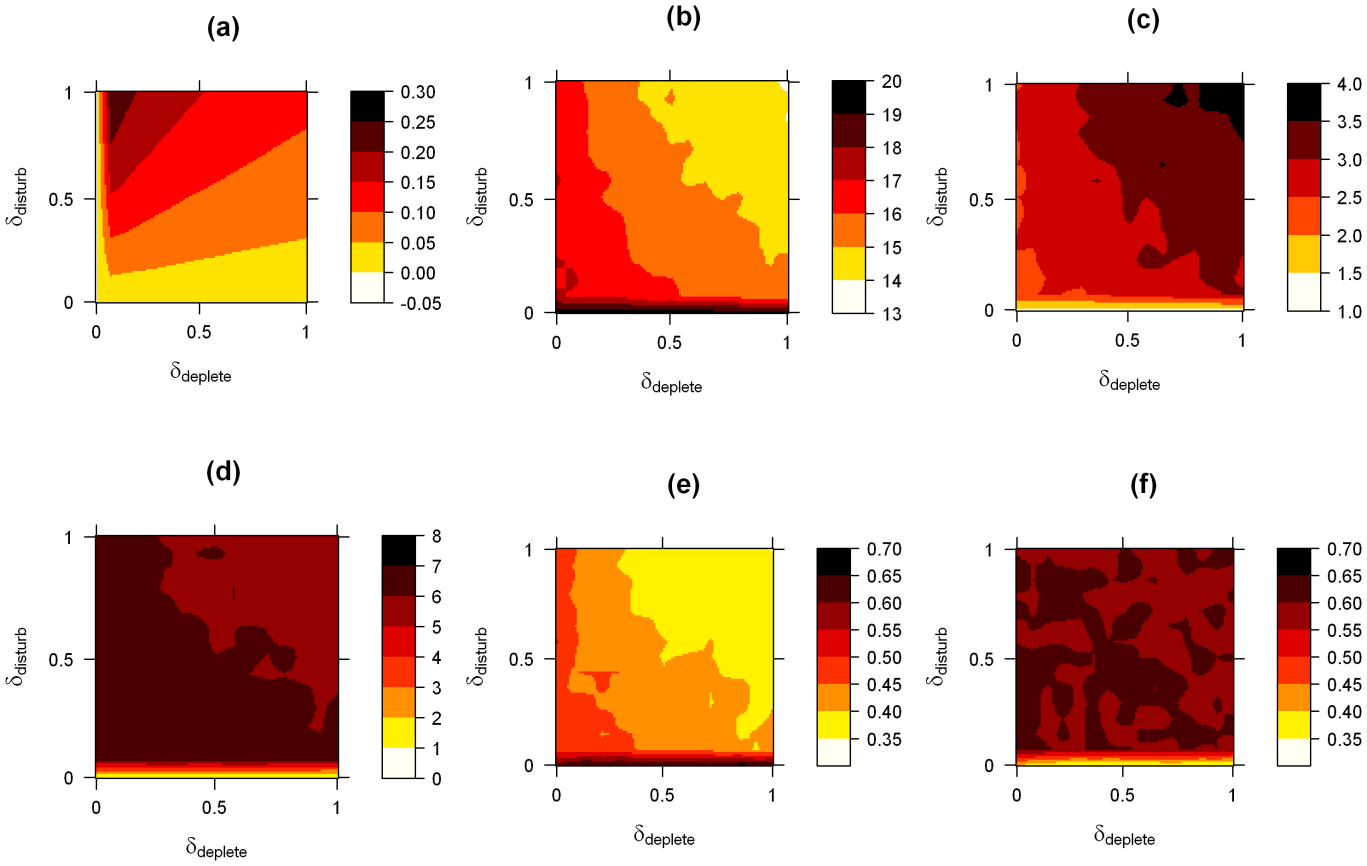
Effect of individual-level interactions with the nutrient fields on the observed dynamics. The effect of individual-level interactions with the nutrient fields on the observed dynamics, starting from a homogeneous underlying nutrient field *U*. We show averages over 100 simulation runs per *(δ_deplete_, δ_disturb_)* parameter combination and simulate *N = 20* individuals. To cover the *(δ_deplete_, δ_disturb_)* parameter space, we simulated combinations of 15 equally spaced values for *δ_deplete_* and *δ_disturb_* between *0* and *1*, resulting in *225* parameter combinations. For a smooth illustration of our simulation results, we interpolated between simulation results to obtain a *(δ_deplete_, δ_disturb_)* grid at an approximate resolution of *0.01* units per cell. All other parameter values are given in table 1. Panel (a) shows the average consumption of individuals, normalised by *δ_deplete_*, (b) shows the number of groups in the population, (c) the size of the largest group, (d) the tortuosity of individual movement, (e) shows the polarisation of the largest group in the population and (f) the angular momentum of the largest group.

To quantify the observations from figure 1b-d, we record the summary statistics described in the methods section and systematically vary *δ_deplete_* and *δ_disturb_* (fig. 2). The normalised consumption of individuals increases with increasing *δ_disturb_*, but decreases for increasing *δ_deplete_* (fig. 2a). It attains its maximum values for low values of *δ_deplete_* and high values of *δ_disturb_*, suggesting that individuals feed more efficiently by attaining the highest consumption per feeding ability (encoded in *δ_deplete_*) for these parameter values. As expected, the absolute consumption of individuals increases with both parameters (figure S2a). We find that the number of separate groups decreases and that the size of the largest group in the population increases when *δ_deplete_* and *δ_disturb_* are increased (fig. 2b and fig. 2c, respectively). This suggests that the level of aggregation in the population increases for higher values of the two parameters. It appears that once a low threshold of *δ_disturb_* is exceeded, the effect of *δ_deplete_* on the number of groups and the size of the largest group is stronger. Initially, groups within the population may form by chance when individuals feed in close proximity because of random initial conditions or noise in individual movement. Comparing the consumption of the largest to the smallest group in the population shows that on average, for most parameter values, individuals in the largest groups obtain more nutrients than individuals in the smallest groups (figure S2b). This suggests that when individuals release additional nutrients in a group, these released nutrients can locally combine to create an attractive potential for individuals outside the group. Local depletion of nutrients, which we investigate in more detail below, offers an alternative mechanism for aggregation. Figure S3b illustrates this reduction of the number of groups over time and figure S3e shows the increase in size of the largest group in the population over time (see also film S2).

Figure 2d shows that the movement of individuals quickly becomes less linear (i.e. more tortuous) as *δ_disturb_* increases, but decreases again as the parameter increases further. The initial increase in tortuosity is a result of the increasingly circular movement of individuals, as seen in figure 1. The decrease in tortuosity can be explained by the fact that for larger parameter values, individuals form on average larger groups which turn in less tight circles because of repulsive interactions between individuals. For a fixed level of *δ_disturb_*, increasing *δ_deplete_* leads to a decrease in tortuosity. We also find that the largest group in the population is only aligned for very low values of *δ_disturb_* but becomes less aligned as *δ_deplete_* and *δ_disturb_* increase (fig. 2e). This can be explained by the increasingly circular movement of individuals and the fact that large rotating or milling groups are not highly aligned. For low values of *δ_disturb_*, the largest group considered in the computation for alignment is typically of size 2 (compare to fig. 2c). These groups are likely to form by chance when the paths of two individuals cross. Repulsive interactions between two individuals can then result in higher alignment (for an explanation, see [32]). Figure 2f shows that apart from for low values of *δ_disturb_*, the largest groups in our simulations showed fairly high angular momentum as a result of the milling movement described above.

**Figure 3:**
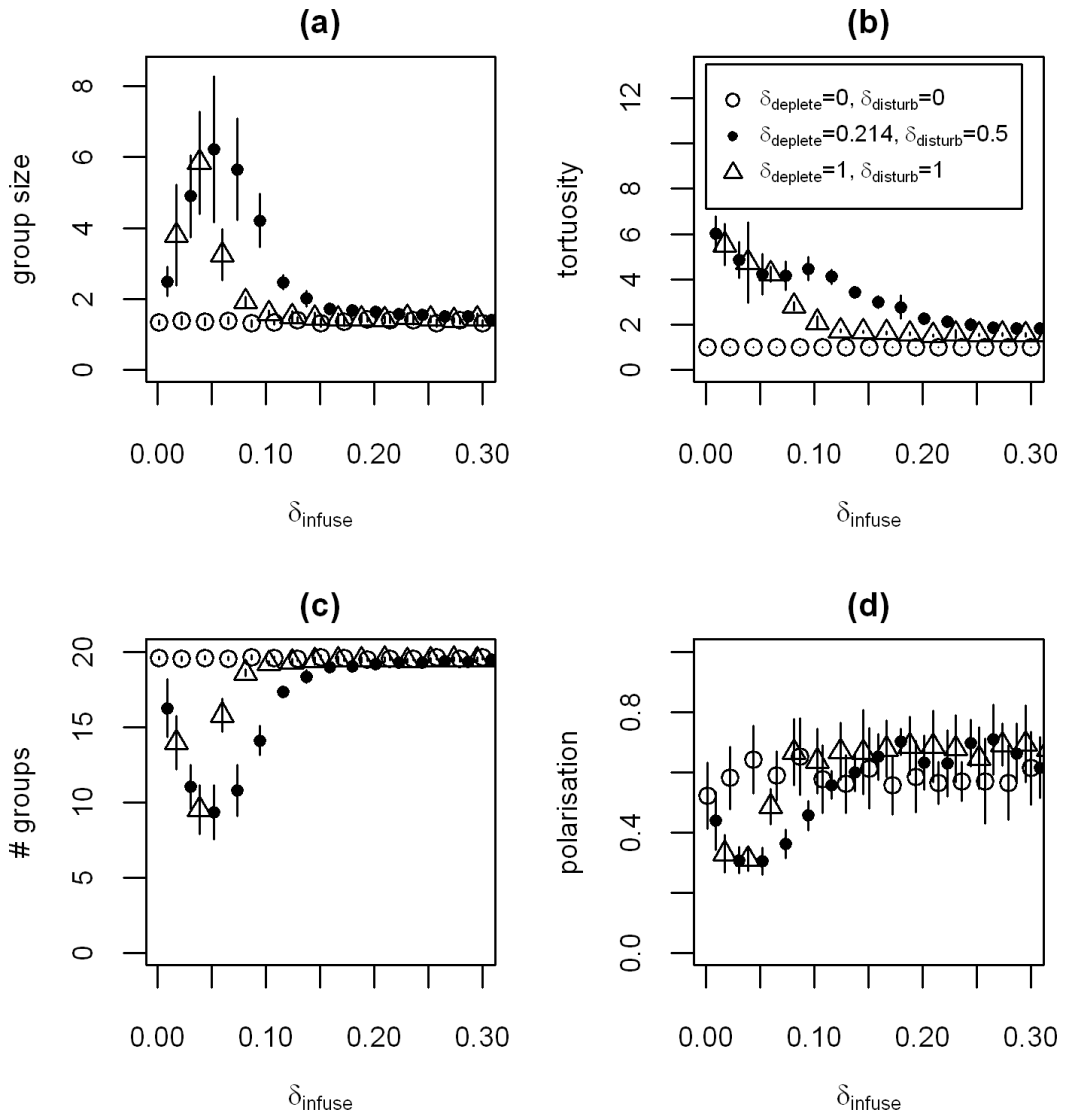
The effect of depleting the nutrient field. We simulate populations of 20 individuals, vary the rate at which nutrients pass from *U* into *Q* (*δ_infuse_*) and start from a homogeneous underlying nutrient field *U*. We show averages over the last 50 seconds of simulation time and report the mean over 10 replicate simulations. We explore three different parameter combinations: *(δ_deplete_, δ_disturb_) = (0, 0) -* white filled circles, *(δ_deplete_, δ_disturb_) = (0.214, 0.5)* - black filled circles and *(δ_deplete_, δ_disturb_) = (1, 1)* - triangles (see also legend in panel b). Values for the three different parameter combinations are offset on the x-axis to ensure error bars (+/−1 s.d.) are visible. All other parameter values are given in the figure or table 1. In panel (a) we show the size of the largest group in the population. In (b) we show the tortuosity of individual movement, in (c) the number of groups in the population and in (d) the polarisation of the largest group in the population.

For the parameters we choose for figures 1 and 2, we find that the values of the summary statistics are stable over the length of our simulations after an initial transitional period (with the exception of the number and size of groups, as discussed above; see figure S3). However, depletion of resources can have an effect on the observed dynamics. To see this, we vary *δ_infuse_*, the rate at which nutrients become available by moving from *U* to *Q* (fig. 3). In combination with high values of *δ_deplete_*, high values of *δ_infuse_* lead to a faster local depletion of nutrients, as both nutrients pools *U* and *Q* get depleted. Consequently, for higher values of *δ_infuse_*, the movement dynamics in the last *50 seconds* of *300 second* simulations become indistinguishable from the movement dynamics when individuals do not interact with the nutrient fields (*δ_deplete_* and *δ_disturb_ = 0*). If *δ_deplete_* is higher, this effect occurs for lower values of *δ_infuse_*. Figure 3a,c show that local depletion of nutrients can be a mechanism resulting in higher levels of aggregation, at least temporarily. This result may appear counter-intuitive at first glance. However, if nutrients are depleted locally in a finite environment, this effectively causes a reduction of the space individuals prefer to occupy. These spatial constraints can then result in a concentration of individuals, as in our simulations. Figure S4 gives examples for how the movement dynamics develop over the course of a *300 second* simulation and comparison to figure S3 is useful to show the relative effect of local nutrient depletion.

**Figure 4:**
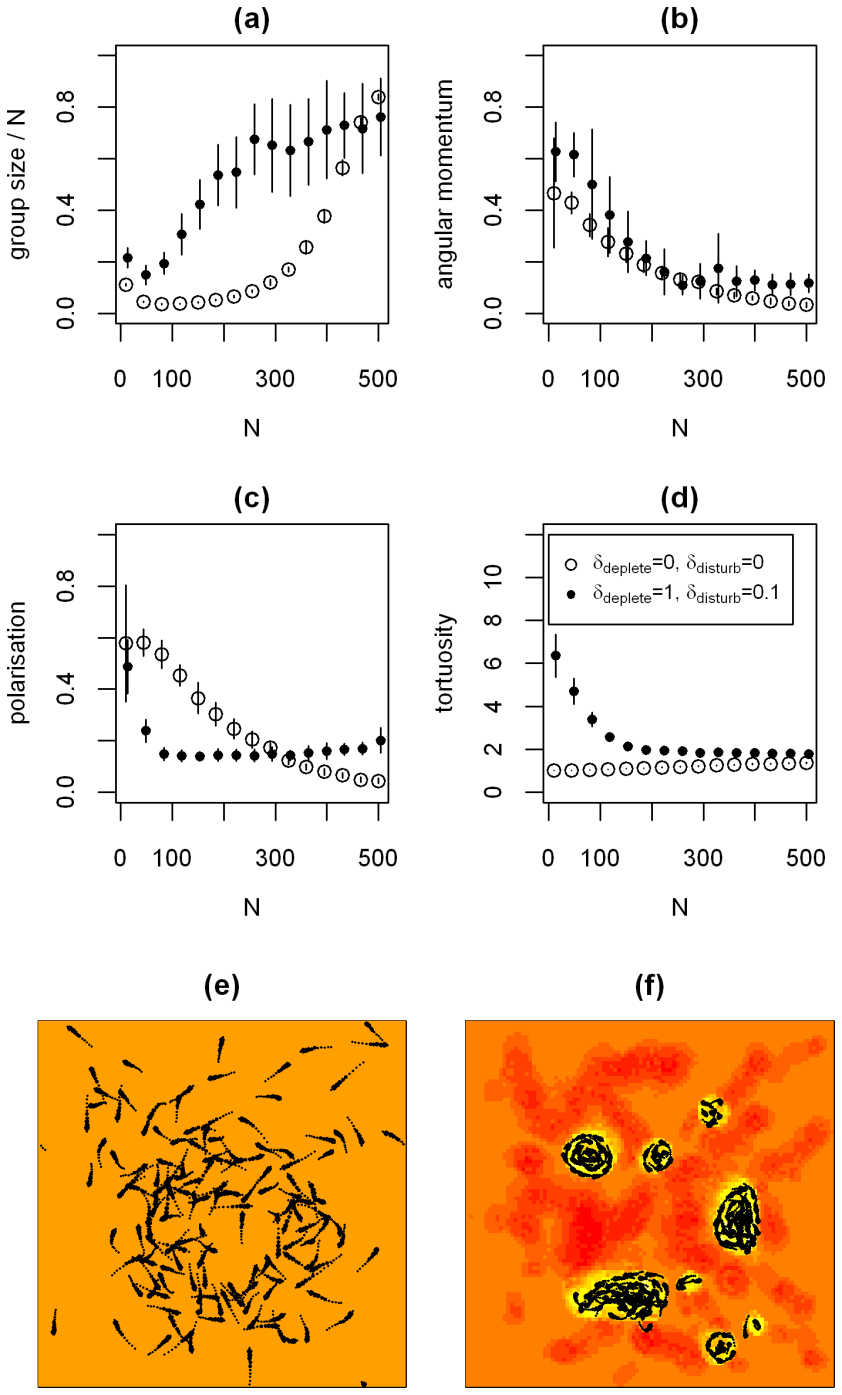
The effect of increasing the population density. In (a-d) we show mean values over 15 simulations. We explore two different parameter combinations: *(δ_deplete_, δ_disturb_) = (0, 0)* - white filled circles and *(δ_deplete_, δ_disturb_) = (1, 0.1)* - black filled circles (see also legend in panel d). Values for the two different parameter combinations are offset on the x-axis to ensure error bars (+/−1 s.d.) are visible. (a) the size of the largest group in the population normalised by the population size *N*. Panel (b) shows the angular momentum of the largest group in the population, (c) shows the polarisation and (d) the tortuosity of individual movement. (e,f) movement dynamics and available nutrient field *Q* for *N = 150* at the end of a *300 second* simulation, showing the last second of each individual trajectory. In (e) we show the case when *(δ_deplete_, δ_disturb_) = (0, 0)* and in (f) we show log values of the available nutrient field *Q* for the case *(δ_deplete_, δ_disturb_) = (1, 0.1).* Brighter colours indicate higher levels of available nutrients in *Q*. The colour scales indicating the level of available nutrients differ between (e) and (f), but since individuals do not interact with the nutrient fields in (e) this does not matter. The darker regions in (f) show that the separate groups move slowly through the environment and ‘graze’ the available nutrients. All simulations start from a homogeneous underlying nutrient field *U*. Other parameters values can be found in the figure or in table 1.

It has been shown that the density of populations can have a strong effect on the movement dynamics that emerge from individual actions (e.g. [33], [34]). Thus far, we have focused on small population sizes (*N = 20*). We now relax this constraint to explore the effect of population density on the observed dynamics. We compare the case when at low population densities individuals move in tight circles, feeding on the nutrients released by their movement (see fig. 1d) with the baseline case when individuals do not interact with the nutrient fields. We first consider the baseline case. As the population density increases, the size of the largest group normalised by the populations size *N* increases nonlinearly (fig. 4a – the decrease for low *N* results from the normalisation). This increase in group size is expected, as we keep the size of the interaction range we use to define groups (*r = 0.3 units*) and the extent of the environment constant. The polarisation and the angular momentum of the largest group decrease and the tortuosity of individuals’ movement slowly increases at a low level in our simulations (fig. 4b-d). The only interactions between individuals in these simulations consist of short-range repulsion. As the population density increases, the frequency of these interactions increases, resulting in more changes in direction and therefore increased tortuosity of individual movement paths. For the case when individuals interact with the nutrient fields, the size of the largest group normalised by the population size *N* increases faster with *N* than in the base-line case, but seems to saturate for large *N*. This suggests that the interactions of individuals with the nutrient fields promote aggregation. The angular momentum of the largest group decreases with *N* (fig. 4b) and the polarisation initially drops, but then increases with increasing population density, albeit at a very low level (fig. 4c). Overall, the tortuosity decreases with increasing population density (fig. 4d). The snapshot of a simulation in figure 4f suggests an explanation for the effect of population density on the summary statistics. For high population densities, large groups that move through the environment form, effectively ‘grazing’ the underlying nutrient fields. These groups display vortex-like, but not necessarily circular (hence the low angular momentum), dynamics that translocate across the environment. The translocation is reflected in increasing levels of alignment of the population and the vortex-like dynamics in large groups reduce the tortuosity of individual movement paths, as repulsive interactions make tight circles more difficult.

## Discussion

We have presented a model for individual movement in a resource landscape, focusing on the effect of individual-to-resource landscape interactions on the observed movement dynamics. Our model suggests possible mechanisms and produces clear predictions for the movement dynamics in resource landscapes with different properties: we expect more tortuous individual movement paths and higher levels of aggregation in populations occupying environments where individual movement makes more nutrients available. We also show how observed movement dynamics could change when local nutrient sources are depleted or when the population density is increased.

**Figure 5:**
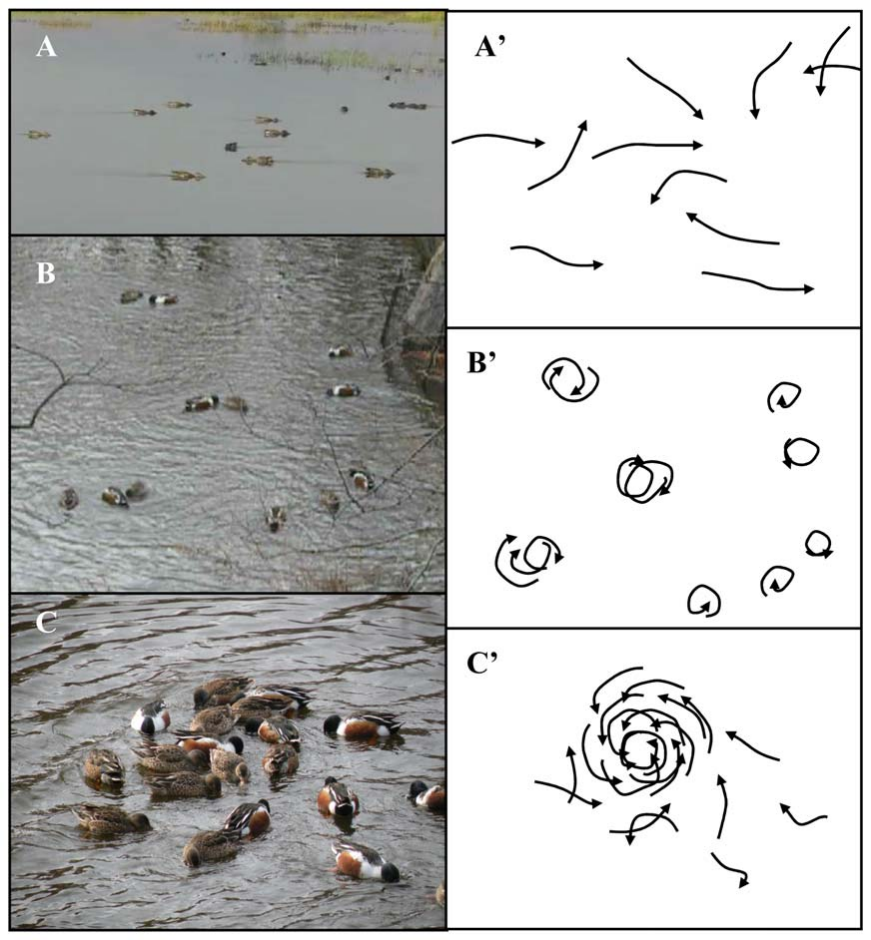
Illustration of three types of feeding behaviour observed in Northern Shovellers (*Anas clypeata*). The left and right hand column show snapshots from video recordings and illustrations of individual movement paths for the corresponding feeding events, respectively. (A, A’) The individual trajectories are approximately linear and individuals are relatively distant from each other. (B, B’) Individual birds and small groups of birds swim in tight circles. (C, C’) A large group of Shovellers swim head to tail in a compact vortex formation. The pictures show that most individuals have their beak in the water and are actively filtering the water, illustrating that nutrients are available close to the water surface. Picture in (A) by Larry Jordan, location: Sacramento National Wildlife Refuge, California, USA, late summer 2011. Pictures in (B,C) by Johann Delcourt, location: Central Park, New-York City, USA, winter 2012.

The motivation for our research is best illustrated with an example. Shoveller ducks (*Anatidae*) are a species of dabbling ducks which are typically found on marshes and in ponds. They are highly specialized for filter-feeding on zooplankton [29], [35], [36]. Shovellers display a range of behaviours whilst filter-feeding (see figure 5). Single birds can be observed to swim in a tight circle and it has been suggested that this movement causes food to rise to the surface allowing the birds to filter it from the water [23]–[25]. However, individual Shovellers also feed whilst moving in a straight line [35], [37]. In addition to feeding individually, Shovellers can also be observed to feed in groups. The pink-eared duck and Australasian Shovellers (*Anas rhynchotis*) tend to feed in small groups of two to five individuals. Individuals swim head to tail in a line or arrowhead formation whilst filtering nutrients from the water [29]. These species also perform a whirling vortex formation in which ducks swim head to tail in a tight circle while filter-feeding (Ibid.). The most impressive feeding vortices with dozens of birds involved can be observed in the Northern Shoveller (*Anas clypeata*; [38]; pers. obs).

To date it is not clear what causes the birds to adopt particular feeding behaviours at different times. Individuals may alter the nature or extent of their social interactions or individuals may consciously adopt particular feeding strategies depending on the context. Comparing figures 1 and 5 suggests that our simulations can reproduce the feeding behaviours observed in Northern Shovellers, but we note that we used a strong attractive nutrient potential to obtain aggregation and vortex-like movement at the population level (fig. 1a). Based on the predictions of our model, we expect filter-feeding ducks to move individually in tight circles in shallow ponds where the birds’ movement disturbs the bottom of the pond and thus frees nutrients. Over time, aggregations could form or, alternatively, patches of high nutrient concentration may cause birds to aggregate and form large and relatively stable feeding vortices. Increased rates of local nutrient depletion could temporarily lead to higher levels of aggregation (fig. 3). In deeper ponds, where the movement of ducks swimming on the surface is less likely to release nutrients from the bottom of the pond, or when the quantity of nutrients released relative to the already available nutrients is low, we expect more linear movement of feeding ducks and generally a lower level of aggregation in populations.

The swirling movement of wadepipers (*Phalaropus sp*., Scolopacidae) in shallow water has been shown experimentally to raise prey items to the surface and therefore within reach of the birds [22]. While phalaropes frequently display this behaviour at freshwater sites, it is never observed when food items are already abundant at the water surface [22], [39]. It remains to be tested whether the swirling feeding behaviour of phalaropes is a by-product of individual-to-resource landscape interactions which we consider here, or a deliberate feeding strategy. We suggest that our predictions could provide a useful starting point for investigating the mechanisms underlying group-level movement phenomena and aggregation in these species.

Tadpoles provide an ideal system to experimentally test our predictions in the laboratory. It has already been shown that spadefoot toad tadpoles (*Spea multiplicata*) form feeding vortices around an initially provided patch of nutrients [20]. These experiments have also revealed that the movement of the tadpoles affects the distribution of food items. We are therefore optimistic that these experiments can be adapted to test our predictions by varying the distribution and initial availability of nutrients. For example, instead of suspending food items in the water, as in [20], food items that sink in water could be used.

We have shown that for some parameter combinations individuals in the largest group have a higher consumption of nutrients than individuals in the smallest group in the population (figure S2). This suggests that individuals can derive a benefit from being close to others. It is possible that individuals could evolve or adapt social interactions to better exploit such scenarios. For example, filter-feeding ducks may deliberately move towards conspecifics that display the above mentioned ‘swirling’ feeding behaviour, exploiting the signal given by the feeding individual. Theoretical work has shown that social interactions in such producer-scrounger systems can lead to fluctuating spatiotemporal patterns in populations feeding on nutrients without releasing them through their movement [40]. In general, social interactions, such as the tendency of individuals to align or move towards each other, can strongly affect the observed movement patterns in animals [28], [34]. While many animals are not capable of long-range social interactions, it is likely that some animals have a repertoire of social interactions that goes beyond short-range repulsive interactions. In such animals social interactions could have a strong effect on the level of aggregation or polarisation in populations, for example. However, the precise mechanisms underlying the feeding behaviours of many species are still unknown. We have deliberately restricted social interactions between individuals to short-range repulsive reactions in our model to highlight the possible role of indirect interactions via stigmergy. We do not wish to suggest that our model fully explains the feeding behaviours observed in different species, but we suggest that it presents a parsimonious starting point for further investigation and that it could occur in conjunction with other mechanisms.

Most of our simulations start from homogeneous nutrient distributions. Figure 1a demonstrates that the distribution of nutrients can have a strong effect on the dynamics we observe. Concentrating all nutrients in one patch presents an extreme case, but it is conceivable how environments with more patches could lead to increased local aggregation within populations. At the individual level, patchy resource distributions may lead to specialised search strategies in feeding animals [2]–[4].

Theoretical work by Grossmann and co-workers [32] has investigated a self-propelled particle model in which individuals only interact through inelastic collisions. This concept is similar to the case when individuals do not interact with the nutrient fields in our model. Simulations of the model by Grossmann et al. [32] showed that the polarisation changes abruptly from very low values to high values as the population density is increased. This contrasts with our findings in fig. 4c. There are a number of possible explanations for this discrepancy. First, the two models are not identical. Grossmann et al. [32] consider normal and tangential forces during collisions rather than simple repulsion within a fixed range. The normal forces are modelled by a spring pulling particles away from each other and therefore depend on the distance between particles. The tangential forces occur when particles overlap and are modelled as friction forces. Second, we run our simulations for a fixed period of time and do not wait until the simulations reach a stable state in which the summary statistics remain approximately constant. We choose this approach to facilitate comparison to simulations in which individuals interact with the nutrient fields and where a stable state may only be reached when all nutrients are depleted. Finally, it is possible that we do not increase the population density to sufficiently high levels for the transition to a highly aligned state to occur. However, detailed model comparison is not the subject and therefore beyond the scope of this research. We suggest that the case when individuals do not interact with the nutrient fields provides a useful baseline for comparison.

Population density is one of the key drivers affecting the movement dynamics in populations (e.g. [33], [34]). While we do not expect that filter-feeding ducks reach population densities in the range we explore in figure 4 [23], it is conceivable that large numbers of tadpoles could be concentrated in small ponds or temporary rain pools. The snapshot of movement dynamics in figure 4f is somewhat reminiscent of the movement and spatial development of bacterial colonies [26], [41]. However, in bacterial colonies other factors, such as energy budgets, chemorepellants and reproduction strongly affect the movement dynamics [26], [41] and we do not wish to suggest our model could apply to such systems. Nevertheless, the similarity between very different systems suggests certain commonalities that could hint at more general underlying principles [7].

The key ingredient of our model is the release of additional nutrients through the movement of individuals. Our implementation is inspired by the feeding behaviour of particular species (tadpoles, filter-feeding ducks, wadepipers), but the concept of individuals causing changes to the distribution of nutrients that may be advantageous for others is more general. For example, juvenile herring (*Clupea harengus*) feed by attacking individual copepods [42]. Copepods are capable of fast avoidance maneuvers [42] and one could imagine that herring following other herring into a patch of copepods could benefit from increased copepod densities on the sides of the attack trajectory of leading herring. Whether our modelling framework could be adapted to investigate such additional scenarios will depend on their particular characteristics and underlying mechanisms. However, we suggest that considering two-way interactions between feeding individuals and resource landscapes could help to explain fine-scale movement dynamics.

## Supporting Information

Film S1 Films S1–S4 show the movement illustrated in figure 1. Films S1–S4 correspond to panels (a)–(d) in figure 1. One second of film corresponds to 2.5 seconds of simulation time.
The distribution of available nutrients, *Q*, is shown in colour. Brighter colours correspond to higher nutrient concentrations in *Q*. Colours do not correspond to absolute nutrient concentrations, but show relative nutrient levels at one point in time. This explains why the patch of high nutrient distribution at the start of film 1 seems to disappear as soon as individuals release additional nutrients through their movement, for example. We choose this way of representing the nutrient field as it is the relative nutrient concentrations and not the absolute values that affect the movement of individuals in our simulations.
**‘Vortex’ around an area of high underlying nutrient concentration.** *(δ_deplete_, δ_disturb_) = (1, 0.1)*. Compare to figure 1a in the main text. Initially and occasionally thereafter, individuals move in opposite directions in the vortex.
(WMV)

Film S2 **‘Swirling individuals’.** *(δ_deplete_,δ_disturb_) = (1, 0.1)*. Compare to figure 1d in the main text. Individuals that are initially close together, form groups. In groups, individuals can benefit from the nutrients released by other group members enabling them to achieve higher consumption rates (). Over time this increased availability of nutrients in groups can attract additional individuals to the group, leading to larger aggregations (e.g. figure S3e).
(WMV)

Film S3 **Dynamics during weak release of nutrients through individuals’ movement.** *(δ_deplete_, δ_disturb_) = (1,0.01)*. Compare to figure 1c in the main text. Note that since *Q* is initially zero everywhere, it takes some time for the nutrients from *U* to get into *Q*, which explains why the dynamics are initially very similar to the ones in film S2.
(WMV)

Film S4 **No release of nutrients through individuals’ movement.** *(δ_deplete_, δ_disturb_) = (1, 0)*. Compare to figure 1b in the main text.
(WMV)

Figure S1 **Speed time series and distribution of individual speeds over 10 seconds of simulated time.** We compute speeds as the displacement of individuals over one simulation step (0.1 seconds). The left hand column shows the speed time series of a randomly chosen individual and the right hand column shows the distribution of individual speeds accumulated over the same time interval and the entire population. All simulations start from a homogeneous underlying nutrient field *U* and we simulate *N = 20* individuals. All other parameter values not stated here can be found in table 1 in the main text. (a,b) *(δ_deplete_, δ_disturb_) = (1,1)*. (c,d) *(δ_deplete_, δ_disturb_) = (1,0.1)*, compare to film S2. (e,f) *(δ_deplete_, δ_disturb_) = (1,0.01)*, compare to film S3. (g,h) *(δ_deplete_, δ_disturb_) = (0,0)*.
(TIF)

Figure S2 **The effect of individual-level interactions with the nutrient fields on consumption.** Simulations start from a homogeneous underlying nutrient field *U*. We show averages over 100 simulation runs and simulate *N = 20* individuals (cf figure 2 in the main text). All other parameter values are given in table 1. Panel (a) shows the average consumption of individuals without the normalisation used in figure 2a in the main text. (b) shows the difference between the average consumption in the largest group and the consumption in the smallest group in the population normalised by the average consumption shown in (a). Note that for *δ_deplete_ = 0*, this measure is not defined. The smallest group could be represented by an isolated individual. Positive values indicate that consumption in the largest group is on average higher than in the smallest group and we find this is the case for most parameter values.
(TIF)

Figure S3 **The development of group dynamics over time.** Simulations start from a homogeneous underlying nutrient field *U*. We show the average over 30 simulation runs. Each data point is an average over 8 seconds of simulation time. Error bars show+/−1 standard deviation. We explore three different parameter combinations: *(δ_deplete_, δ_disturb_) = (0, 0)* in grey, *(δ_deplete_, δ_disturb_) = (0.214, 0.5)* in blue and *(δ_deplete_, δ_disturb_) = (1, 1)* in red (see also legend in panel b). (a) polarisation of the population, (b) number of groups formed in the population, (c) tortuosity across individuals, (d) polarisation of the largest group in the population, (e) size of the largest group in the population and (f) absolute consumption across individuals.
(TIF)

Figure S4 **The development of group dynamics over time with significant nutrient depletion.** Simulations start from a homogeneous underlying nutrient field *U* with an increased infusion rate from *U* into *Q* (*δ_infuse = _0.06*). We show the average over 30 simulation runs. Each data point is an average over 8 seconds of simulation time. Error bars show+/−1 standard deviation. As in figure S3, we explore three different parameter combinations: *(δ_deplete_, δ_disturb_) = (0, 0)* in grey, *(δ_deplete_, δ_disturb_) = (0.214, 0.5)* in blue and *(δ_deplete_, δ_disturb_) = (1, 1)* in red (see also legend in panel b). The panels show the same summary statistics as in figure S3. For example for *(δ_deplete_, δ_disturb_) = (0.214,0.5)* there appears to be an effect of the local depletion of nutrients which is visible in the size of the largest group which increases and appears to reach a peak after about 250 seconds after which it starts to decline.
(TIF)
