# Supplementary figures and images for: Individual-to-Resource Landscape Interaction Strength Can Explain Different Collective Feeding Behaviours
Source: PLoS One. 2013 Oct 9;8(10):e75879. doi: 10.1371/journal.pone.0075879 (PMC3794026; doi:10.1371/journal.pone.0075879)

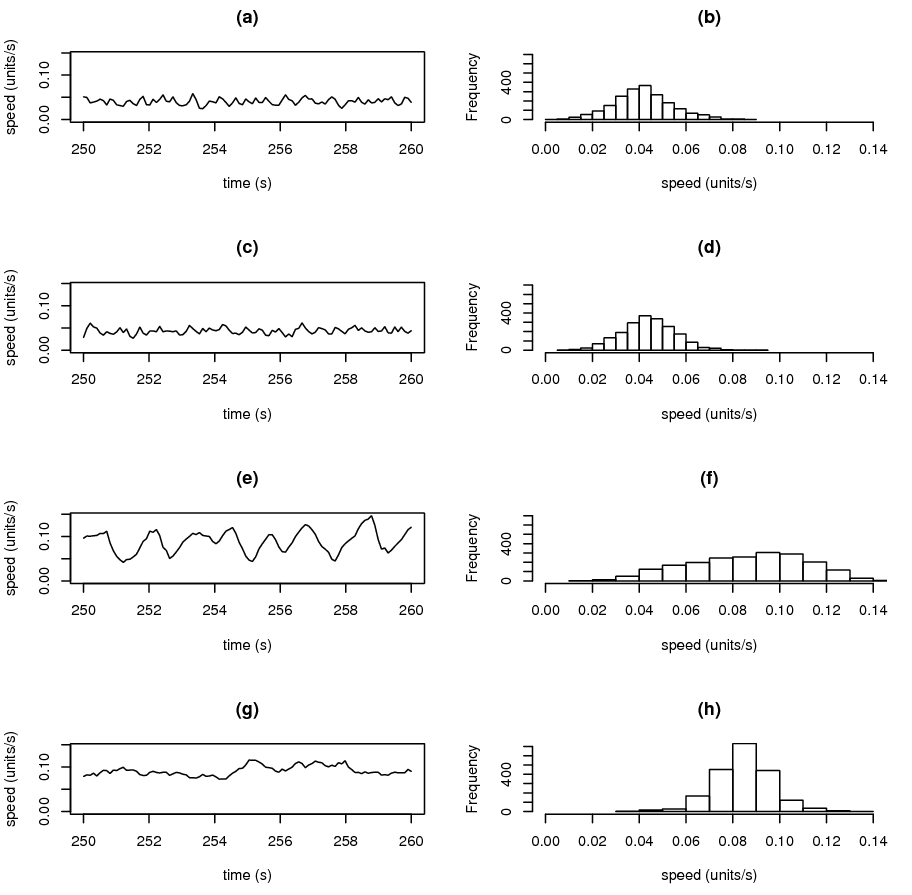

Supplement: Figure S1 — Speed time series and distribution of individual speeds over 10 seconds of simulated time. We compute speeds as the displacement of individuals over one simulation step (0.1 seconds). The left hand column shows the speed time series of a randomly chosen individual and the right hand column shows the distribution of individual speeds accumulated over the same time interval and the entire population. All simulations start from a homogeneous underlying nutrient field U and we simulate N = 20 individuals. All other parameter values not stated here can be found in table 1 in the main text. (a,b) (δdeplete, δdisturb) = (1,1). (c,d) (δdeplete, δdisturb) = (1,0.1), compare to film S2. (e,f) (δdeplete, δdisturb) = (1,0.01), compare to film S3. (g,h) (δdeplete, δdisturb) = (0,0). (TIF) [file pone.0075879.s005.tif]

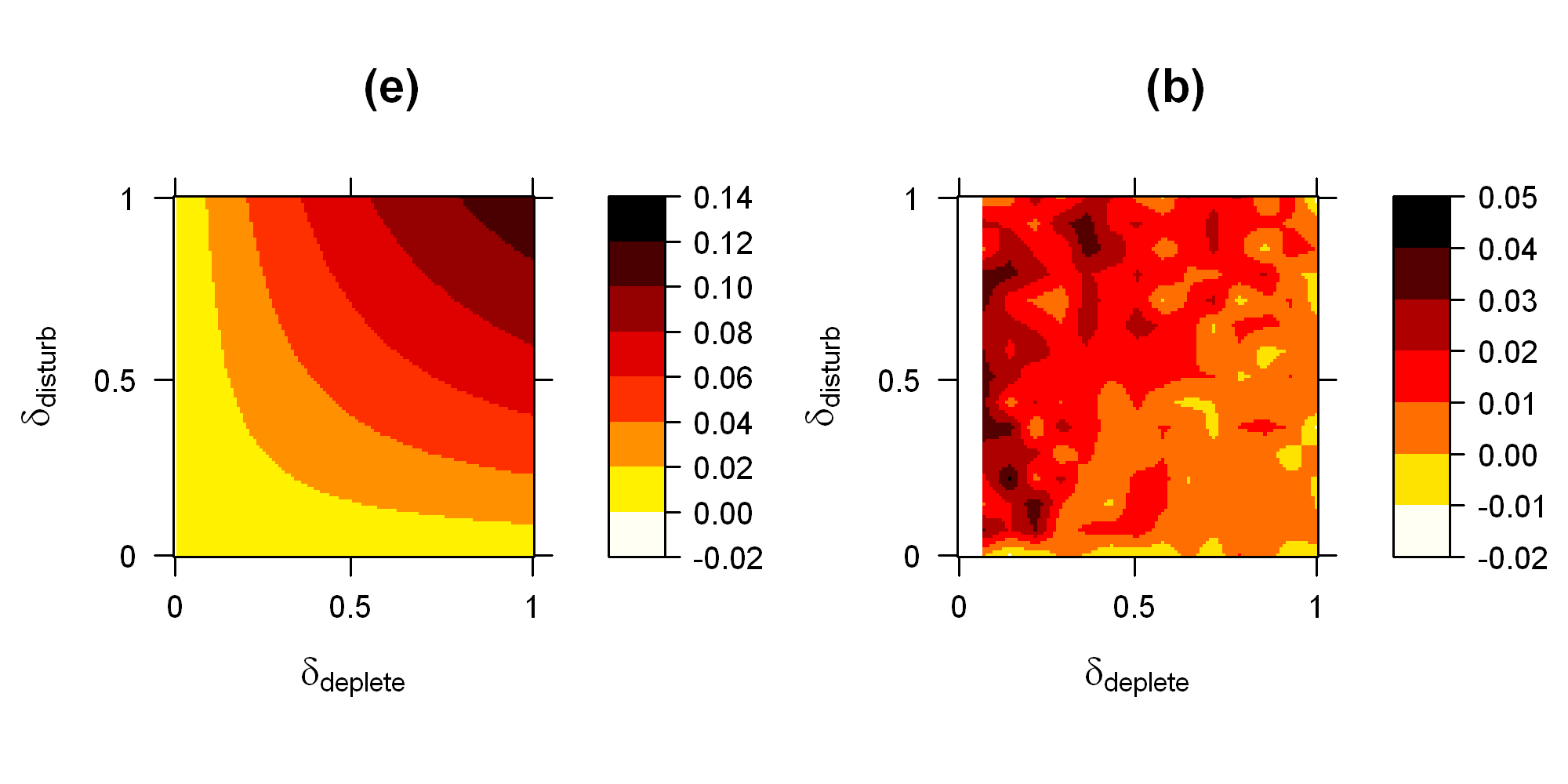

Supplement: Figure S2 — The effect of individual-level interactions with the nutrient fields on consumption. Simulations start from a homogeneous underlying nutrient field U. We show averages over 100 simulation runs and simulate N = 20 individuals (cf figure 2 in the main text). All other parameter values are given in table 1. Panel (a) shows the average consumption of individuals without the normalisation used in figure 2a in the main text. (b) shows the difference between the average consumption in the largest group and the consumption in the smallest group in the population normalised by the average consumption shown in (a). Note that for δdeplete = 0, this measure is not defined. The smallest group could be represented by an isolated individual. Positive values indicate that consumption in the largest group is on average higher than in the smallest group and we find this is the case for most parameter values. (TIF) [file pone.0075879.s006.tif]

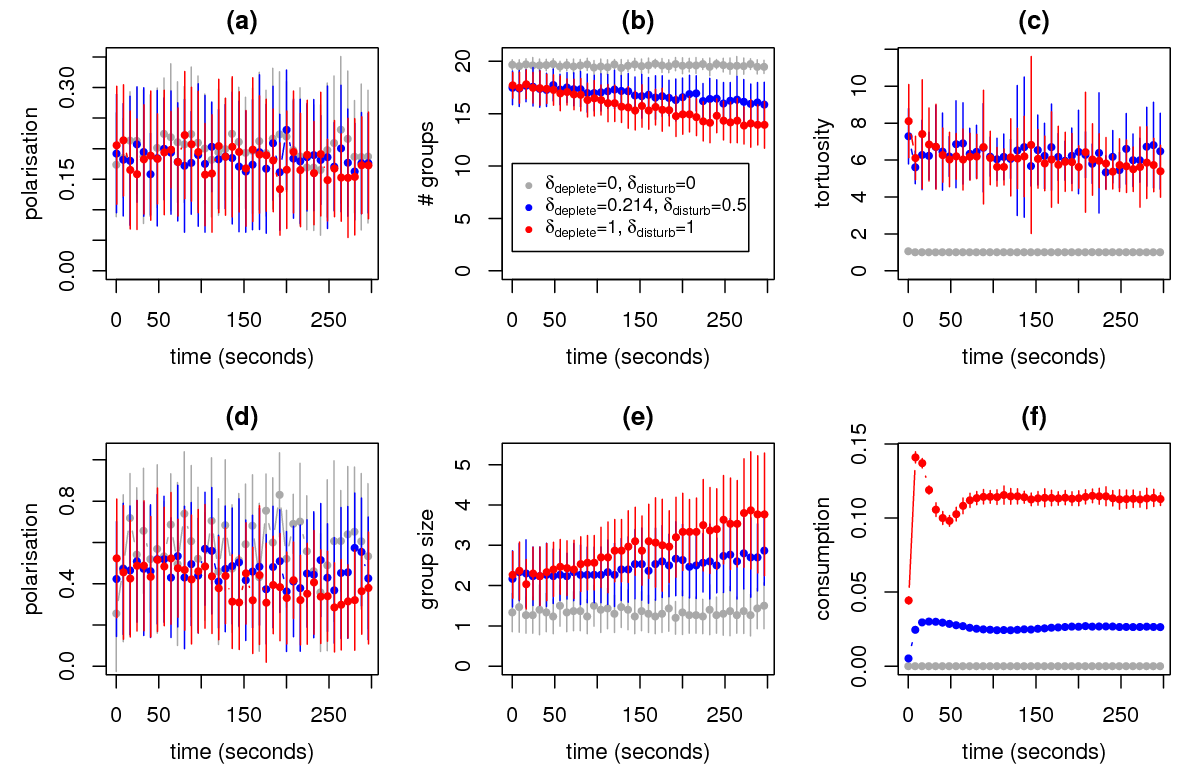

Supplement: Figure S3 — The development of group dynamics over time. Simulations start from a homogeneous underlying nutrient field U. We show the average over 30 simulation runs. Each data point is an average over 8 seconds of simulation time. Error bars show+/−1 standard deviation. We explore three different parameter combinations: (δdeplete, δdisturb) = (0, 0) in grey, (δdeplete, δdisturb) = (0.214, 0.5) in blue and (δdeplete, δdisturb) = (1, 1) in red (see also legend in panel b). (a) polarisation of the population, (b) number of groups formed in the population, (c) tortuosity across individuals, (d) polarisation of the largest group in the population, (e) size of the largest group in the population and (f) absolute consumption across individuals. (TIF) [file pone.0075879.s007.tif]

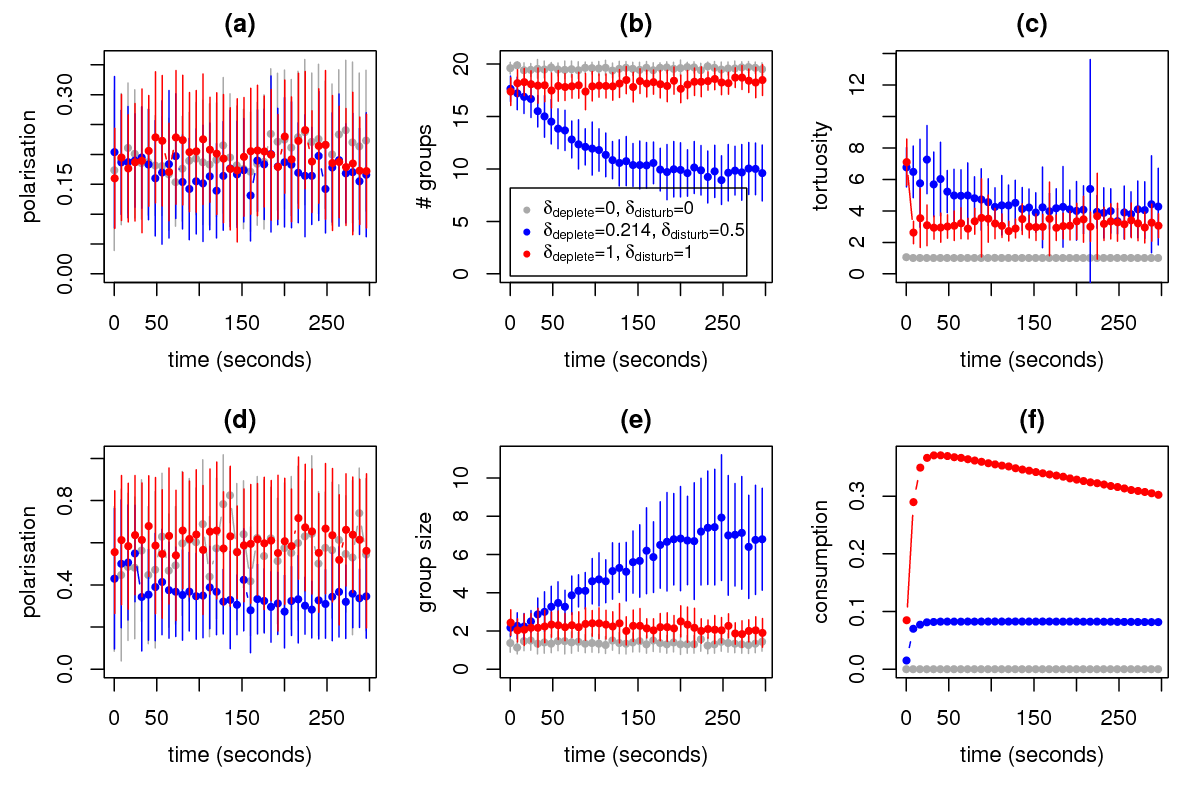

Supplement: Figure S4 — The development of group dynamics over time with significant nutrient depletion. Simulations start from a homogeneous underlying nutrient field U with an increased infusion rate from U into Q (δinfuse = 0.06). We show the average over 30 simulation runs. Each data point is an average over 8 seconds of simulation time. Error bars show+/−1 standard deviation. As in figure S3, we explore three different parameter combinations: (δdeplete, δdisturb) = (0, 0) in grey, (δdeplete, δdisturb) = (0.214, 0.5) in blue and (δdeplete, δdisturb) = (1, 1) in red (see also legend in panel b). The panels show the same summary statistics as in figure S3. For example for (δdeplete, δdisturb) = (0.214,0.5) there appears to be an effect of the local depletion of nutrients which is visible in the size of the largest group which increases and appears to reach a peak after about 250 seconds after which it starts to decline. (TIF) [file pone.0075879.s008.tif]
